# Risk factors of vitamin D deficiency among 15-year-old adolescents participating in the Malaysian Health and Adolescents Longitudinal Research Team Study (MyHeARTs)

**DOI:** 10.1371/journal.pone.0200736

**Published:** 2018-07-19

**Authors:** Shiao Wei Quah, Hazreen Abdul Majid, Nabilla Al-Sadat, Abqariyah Yahya, Tin Tin Su, Muhammad Yazid Jalaludin

**Affiliations:** 1 Department of Paediatrics, Faculty of Medicine, University Malaya, Kuala Lumpur, Malaysia; 2 Centre for Population Health (CePH), Department of Social and Preventive Medicine, Faculty of Medicine, University Malaya, Kuala Lumpur, Malaysia; 3 Julius Centre University Malaya (JCUM), Department of Social and Preventive Medicine, Faculty of Medicine, University Malaya, Kuala Lumpur, Malaysia; Garvan Institute of Medical Research, AUSTRALIA

## Abstract

**Background:**

This study is to determine the prevalence and risk factors of vitamin D deficiency (vitamin D ≤ 50 nmol/L) among 15-year-old Malaysian adolescents. By identifying potential risk factors, prevention strategies and interventions can be carried out to improve the vitamin D status in adolescents.

**Methods and findings:**

Stratified random sampling design was used to select adolescents from 15 urban and rural secondary schools in Selangor, Perak and Kuala Lumpur, Malaysia. Data collection was carried out from 1^st^ April 2014 to 30^th^ June 2014. Information regarding socio-demographic characteristics, sun exposure and sun protective behaviours, clinical data and environmental factors were collected. Blood for total vitamin D was sampled. Descriptive and multivariate logistic regressions were performed. Total 1061 participants were analyzed (62% were female; mean age 15.1 ± 0.4 years). The prevalence of vitamin D deficiency was 33%. Mean vitamin D was lower in female (53 ± 15 nmol), obese (body fat percentage (≥25%^m^; ≥33.8%^f^) (56 ± 16 nmol/L), Malays (58 ± 18 nmol/L) and Indians (58 ± 15 nmol/L). In multivariate analysis, female (OR = 5.5; 95% CI: 3.4–7.5), Malay (OR = 3.2; 95% CI: 1.3–8.0), Indian (OR = 4.3; 95% CI: 1.6–12.0) and those always wearing long sleeve (OR = 2.4; 95% CI: 1.1–5.4) were more likely to have vitamin D deficiency. For female participants, ethnicity {Malays (OR = 6.7; 95% CI: 2.0–18.5), Indian (OR = 4.5; 95% CI: 1.8–19.3)} was an important risk factors. Cloud cover, school residence, skin pigmentation, sun-exposure and sun-protective behaviours were not significant risk factors. The limitation of this study was recall bias as it relied on self-reported on the sun exposure and protective behaviours. The diet factors were not included in this analysis.

**Conclusions:**

The prevalence of Vitamin D deficiency among Malaysian adolescents was considerable. Gender, ethnicity and clothing style were important risk factors.

## Introduction

Vitamin D deficiency among children and adolescents is now recognized as a significant global health issues [1–2]. According to Vitamin D Supplemental Guidelines by Pludowski [3], vitamin D level ≤ 50 nmol/L (vitamin D deficiency) in adolescents have the clinical implications that increased the risk for bone health related problems and cardiovascular diseases in adult [4]. In Malaysia, the prevalence of vitamin D deficiency (vitamin D ≤ 50 nmol/L) in children is high, ranging from 47 to 75%[5–7], despite the fact that Malaysia is a tropical country (latitude 3.13°N, 101.7°E) with ample sunshine throughout the year.

Around 95% of vitamin D is obtained through cutaneous synthesis in the presence of sunlight while the remainder is gained from the diet [8]. Several studies have analyzed the cutaneous factors that affect the presence of vitamin D in adults. These factors include skin pigmentation[9–10] as well as sun-exposure factors such as timing[11–12], duration of exposure[13–14], percentage of exposed skin area[14] and sun-protective behavior[15–17]^.^ Other studies have shown that environmental factors such as increased cloud cover[18], air pollutants[19], winter season[20–21] and higher latitude[22] reduce the effective ultraviolet B radiation that reaches the earth, which thus reduces vitamin D synthesis. Demographic factors such as gender and ethnicity [23–24] have also been found to influence the level of vitamin D. The amount of adipose tissue [25] in the body also affects the vitamin D level, as it has been shown that obese individuals tend to have a lower vitamin D level. With regards to vitamin D from diet, the average dietary intakes of calcium and vitamin D in majority adolescents in Malaysia did not meet the Recommended Nutrient Intake (RNI) [26].

Vitamin D deficiency during adolescence impairs the acquisition of peak bone mass at the end of skeletal growth and maturation, thereby increasing the risk of osteoporosis and the development of various chronic diseases, including cardiovascular, Type 2 Diabetes Mellitus [27] and increases the risk of dental caries [28]. Vitamin D has the immunomodulatory capacity to combat bacteria, reduces the risk of acute respiratory tract infections [29] and prevents the inflammatory and autoimmune diseases in later life [30]. Vitamin D deficiency is also associated with early menarche which increased the risk of cardiometabolic diseases and cancer [31–32]. Vitamin D deficiency also increases the risk of preterm birth [33].There are only a limited number of studies that have attempted to fully elucidate the role of each of the above-mentioned factors in regulating the level of vitamin D in adolescents, especially in the tropics. Therefore, this study was undertaken to determine the prevalence and risk factors of vitamin D deficiency among Malaysian adolescents. By identifying potential risk factors, prevention strategies and interventions can be carried out to improve the vitamin D status in adolescents.

## Methods

### Ethics statement

The study received ethical approval from the Medical Ethics Committee, University Malaya Medical Centre (MEC Ref. No: 896.34). Written informed consent was obtained from parent or guardian and all participating children were also required to sign an assent form. All testing was performed in accordance with the approved guidelines.

### Study design

This study is the first follow-up visit of a prospective cohort study, i.e. the Malaysian Health and Adolescents Longitudinal Research Study (MyHeARTs) which aims to study the risk factors for chronic non-communicable diseases among adolescents, as described previously [34–35]. The study was conducted in the central and northern zone of Peninsular Malaysia, which is 3.13° N of the equator, specifically in Selangor, Perak and the Federal Territory of Kuala Lumpur (WPKL).

The current study aimed to recruit all the male and female Year 3 secondary school students (14–15 year-olds) studying in 15 randomly selected public schools in the above three states. Data collection was carried out from 1^st^ April 2014 to 30^th^ June 2014. Boarding, religious, and vernacular schools were excluded because these schools are not representative of the type of Malaysian schools that the majority of students attend.

The study design and sampling was discussed in previously published articles [7, 34–35]. The only difference is that all Year 3 students in the 15 secondary schools were invited to participate in the study compared to Year 1 students during the first visit to these schools [7, 34]. Written information about the study and consent forms were given to the participants and their parents/guardians. The information sheet included details about the study which helped the students and their parents to make a decision about whether to participate in the study. The participants had to be able to read and understand the national language, Malay and be willing to participate voluntarily. To participate in the study, the students had to attend school during the study day and hand in a signed written informed consent and assent form. Each participant was given a unique identification number to ensure anonymity.

Participants were excluded from the study for the following reasons: (1) refused to take part and without parental consent; (2) had a current endocrine disorder (e.g.: hypoparathyroidism, hyperparathyroidism, rickets, osteoporosis, diabetes mellitus); (3) were on any prescription medications that affect bone metabolism[36] (oral/parenteral steroid, anti-epileptic, anti-oestrogens, anti-retroviral drugs, cytotoxic agents); (4) had a systemic medical illness (e.g.: chronic kidney disease, end-stage liver disease, malabsorption disease, malignancy).

The total sample size required for this study was about 1200, which was calculated by using the formula n = (z^2^.p.q/r.e^2^) x Deff, whereby Deff is design effect (2), z is the standard normal deviate set at 1.96 at 5% level for two-tailed test, p = estimated prevalence, q = 1-p, r = response rate and e = precision level, 72% was used as the estimated prevalence of vitamin D deficiency in children according to the Khor GL study [5]. R (response rate) was 50% based from the first part of MyHeARTs study which was conducted in October to November 2011 [34–35].

### Questionnaire and anthropometric measurements

Socio-demographic information such as gender, age, ethnicity and school location were collected from the self-administered questionnaires. Another questionnaires regarding sun-exposure, was adapted from the Measures of Sun Exposure and Sun Protection Practices for Behavioral and Epidemiologic Research [37]. The participants were asked to complete the questionnaire (S1 File), which aimed to elicit information about their skin pigmentation based on the Fitzpatrick Scale [38] (type 1: very fair, pale white, often freckled; type 2: fair white skin; type 3:light brown; type 4: moderate brown; type 5: dark brown and type 6: deeply pigmented, dark brown to black), the duration spent outdoors during weekdays and the weekend (≤ 30 minutes, 30–60 minutes, 1–2 hours and >2 hours per day), the timing of outdoor activities (7am-10am, 10am-2pm, 2pm-4pm, and 4pm-7pm), body surface area exposed to sunlight (fully covered, so that only both the hands and feet are exposed, wearing short sleeves which also expose hands, arms and forearms; wearing short sleeved T-shirt and shorts, which also exposes legs; or only wearing shorts, which also exposes the torso).

The questionnaire was also designed to examine the sun-protective behaviour of the participants i.e. using sunscreen (any form of topical product such as cream, gel or spray that absorbs or reflects sun’s ultraviolet radiation) [39], wearing a long-sleeved shirt, staying in the shade or under an umbrella, wearing a hat or veil, wearing sunglasses. The questionnaire was assessed for face validity and test reliability by administering it to 50 adolescents (not included in this study) prior to the start of data collection.

As for the anthropometric measurements, body fat percentage was measured by using a Tanita Portable Body Composition Analyzer SC-240 MA and expressed as a percentage (a method validated for Malaysian children by a previous study [40]).

### Biochemical measurements

Blood samples were collected in plain tubes by certified phlebotomists after an overnight fast of at least ten hours. The samples were temporarily stored at four degrees Celsius in a cool box immediately after the blood was taken and then sent to an International Organization for Standardization (ISO) certified hospital pathology laboratory for 25-hydroxyvitmain D(25(OH)D) and intact parathyroid hormone (PTH) analyses. 25-hydroxyvitmain D(25(OH)D) was analyzed using an ADVIA Centaur vitamin D assay, which is an automated direct competitive chemiluminescent immunoassay that detects 25-hydroxy vitamin D (25(OH) D) in serum or plasma. This assay uses a proprietary releasing reagent and a monoclonal antibody. The lower limit of detection of the assay was 3.2 ng/mL (8.0 nmol/L). The inter- and intra-assay coefficients of variability for the lower level mean of 13.6 ng/mL (34 nmol/L) were 11.9 and 4.7% respectively. The inter- and intra-assay coefficients of variability for the upper level mean of 114.1 ng/mL (285.2 nmol/L) were 4.2 and 3.0% respectively.

For intact parathyroid analysis, the laboratory used the ADVIA Centaur intact parathyroid assay which is a two-site sandwich immunoassay that employ direct chemiluminometric technology which uses constant amounts of an antihuman parathyroid antibody in the lite reagent and an antihuman parathyroid antibody in the solid phase reagent. It measures intact parathyroid concentrations up to 1900 pg/mL (201 pmol/L) with a minimum detectable concentration (analytical sensitivity) of 2.5 pg/mL (0.265 pmol/L). The inter- and intra-assay coefficients of variability for the lower level mean of 40.4 pg/mL (4.3 pmol/L) were 5.2 and 5.8% respectively. The inter- and intra-assay coefficients of variability for the upper level mean of 859.3 pg/mL (91.1 pg/mL) were 3.5 and 2.8% respectively.

### Definitions

No consensus has been achieved for the reference intervals for the vitamin D status in adolescents. For this study, based on clinical implications to the skeletal health disease and extra-skeletal health diseases described in the Vitamin D supplement guidelines by Pludowski et al [3], a serum 25-hydroxyvitamin D level ≤ 50 nmol/L was defined as vitamin D deficiency.

The cut-offs for the body fat percentage were set at the 2^nd^, 85^th^ and 95^th^ centiles for “underfat”, “normal”, “overfat” and “obese” respectively for both the male and female participants according to the body fat reference curve for children [41].

The information on the cloud cover of the 15 selected schools during the data collection period were obtained from the local weather station from the Malaysian Meteorological Department. The sky conditions are estimated in terms of how many eights of the sky are covered in cloud, ranging from 0 octa (completely clear sky) through to 8 octa (completely overcast).

### Statistical analysis

All data including socio-demographic data, anthropometric measurements and biochemical measurements were entered into and analyzed using the SPSS Statistics for Windows program, version 22.0, released in 2013 (Armonk, NY, USA). Categorical variables (gender, ethnicity, place of residence, body fatness) were described as frequencies and percentages. The associations between categorical data were measured by Chi-square statistics. The association between the outcome (mean vitamin D) and the determinants was assessed using the Student’s t-test and ANOVA. For the significant results in ANOVA, post hoc tests (Tukey) were also performed. The measure of effect (odd ratio, OR) and 95% confidence interval (95% CI) between the potential risk factors and the outcome, vitamin D deficiency were calculated by performing univariate logistic regression. Respective risk factors that had p-value <0.25 were further analyzed by using multivariable logistic regression [42]. All statistical tests were conducted at the 5% significance level.

## Results and discussions

Initially, 1234 participants were enrolled in the present study. After excluding incomplete data and missing values, the total number of participants eligible for analysis was 1016 with a mean age of 15.1 ± 0.4 (range 14.56 to 16.60) years. No participant had a current endocrine disorder (e.g.: hypoparathyroidism, hyperparathyroidism, rickets, osteoporosis, diabetes mellitus), taking medications that affect bone metabolism or had a systemic medical illness (e.g.: chronic kidney disease, end-stage liver disease, malabsorption disease, malignancy). The total number of participants in the present study was slightly lower than the study executed in 2012 and is described in detail by Majid et al 2016 [35].

### Socio-demographic

The majority of the participants were female, 62% (n = 631). Overall, 338 participants had vitamin D deficiency giving the prevalence of 33%. For female participants, 47% (n = 296) had vitamin D deficiency. Females also had a significantly lower mean serum vitamin D (53 ± 15 nmol/L) than the males (70 ± 16 nmol/L, p < 0.001).

As regards to ethnicity, 83% (n = 846) of the participants were Malay, followed by Indian 8% (n = 81), Chinese 7% (n = 73) and other ethnicities 2% (n = 16). The mean vitamin D level among Chinese participants (69 ± 15 nmol/L, p<0.001) was significantly higher than that among Malay (58 ± 18 nmol/L) and Indian (58 ± 15 nmol/L) participants. There was no statistically significant difference in the mean vitamin D level between Malay and Indian participants.

About 25% (n = 250) of the participants were obese [measured by body fat percentage (≥25%^m^; ≥33.8%^f^)]. The mean vitamin D level was significantly lower (56 ± 16 nmol/L, p < 0.001) in the obese group than in the non-obese group.

### Cutaneous factors

Most of the participants had type 2 to type 4 skin pigmentation (27% type 2: fair white skin; 38% type 3: light brown and 26% type 4: moderate brown). Although Type 4 and Type 5 skin pigmentation had higher mean vitamin D level, they were not statistically significant (p = 0.19). (Table 1).

**Table 1:** Mean 25-hydroxyvitamin D level of Malaysia adolescents according to cutaneous factors and sun-exposure factors.

Data	Number (%)	Mean Vitamin D (nmol/L)	p value
**Type of Skin Pigmentation**			0.19*
Type1- Very fair, pale white	32 (3)	56 ± 18	
Type 2- Fair white skin	269 (27)	58 ± 17	
Type 3- Light brown	389 (38)	59 ± 18	
Type 4- Moderate brown	268 (26)	60 ± 18	
Type 5 –Dark brown	51 (5)	64 ± 16	
Type 6- Deeply pigmented, dark brown to black	5 (1)	54 ± 15	
**Skin Area most Commonly Exposed**			< 0.001**
Only exposed hands & face	401 (40)	52 ± 15	
Wears short sleeves	424 (42)	63 ± 17	
Wears shorts and T-shirt	178 (18)	68 ± 17	
Only wears shorts	13 (1)	63 ± 16	
**Timing of Outdoor Activities (n = 858)**			0.09**
7am-10am	152 (18)	58 ± 18	
10am-2pm	61 (7)	61 ± 16	
2pm-4pm	183 (21)	56 ± 17	
4pm-7pm	462 (54)	60 ± 17	
**Duration of Outdoor Activities During Weekdays**			0.002**
≤ 30 min	272 (27)	56 ± 16	
31min-1 hours	188 (19)	60 ± 18	
1–2 Hours	250 (5)	61 ± 17	
>2 hours	306 (30)	61 ± 19	
**Duration of Outdoor Activities During Weekend**			< 0.001**
≤ 30 min	145 (14)	55 ± 18	
31 min- 1hour	202 (20)	57 ± 28	
1–2 hours	261 (26)	60 ± 19	
> 2 hours	408 (40)	61 ± 17	

*Student T-test

**ANOVA test

### Sun-exposure and sun-protective behaviour

About 75% (n = 645) of the participants spent time outdoors after 2pm (i.e. after school). Only 7% (n = 61) of participants spent their time outdoors from 10am to 2pm, which is the period with the greatest potential for vitamin D synthesis. However, their mean value of vitamin D was not significantly higher compared to others. There was no significant different between males and females in the timing of outdoor activities as well.

During weekdays, 30% (n = 306) of the participants spent more than 2 hours pursuing outdoor activities and this number increased to 40% during the weekend. The mean vitamin D level among those who spent more than 2 hours participating in outdoor activities during weekdays (61 ± 19 nmol/L, p = 0.002) and the weekend (61 ± 17 nmol/L, p<0.001) was significantly higher than among those who spent less than 30 minutes doing so (56 ± 16 nmol/L) during weekdays and the weekend (55 ± 18 nmol/L).

About 40% (n = 401) of the participants wore concealing clothes that only exposed their hands and feet and this group had the lowest mean vitamin D (52 ± 15 nmol/L) as determined by one-way ANOVA (F = 51.87, p value <0.001) compared to those who wore a short-sleeved T-shirt and shorts (68 ± 17nmol/L) (Table 1).

### Environmental factors

Participants from the rural schools (n = 613) had a higher mean vitamin D level (61 ± 18 nmol/L, p = 0.006) than the urban schools (n = 403) despite higher mean cloud cover in the rural area (7.0 ± 0.1 vs 6.9 ± 0.4 octa, p = < 0.001). This may be due to the higher number of female participants in urban schools (64.7%) compared to rural schools (55.7%) (p = 0.001), as well as female tend to wear more concealing clothing.

Further analysis did not reveal a statistically significant difference between urban and rural participants in terms of skin pigmentation, duration of outdoor activities and type of clothing (results not shown).

### Univariate and multivariate analysis

The univariable analysis found that the following were associated with vitamin D deficiency: female (OR = 7.2; 95% CI:5.1–10.3), Malay (OR = 3.9; 95% CI:2.1–7.5) and Indian (OR = 3.4; 95% CI:1.5–7.4), obesity (OR = 1.6; 95% CI: 1.2–2.1), timing of outdoor activities from 7am to 10am (OR = 2.1; 95% CI:1.1–4.1) and from 2pm to 4pm (OR = 2.2; 95% CI: 1.1–4.2), less than 30 minutes of outdoor activities during weekdays (OR = 1.4; 95% CI: 1.0–2.0) and weekend (OR = 2.6; 95% CI: 1.8–3.9), and exposed hands and feet (OR = 3.4; 95% CI: 1.9–4.5) and wearing long sleeves (OR = 4.4; 95% CI: 2.6–7.5). However, the multivariable analysis revealed that, only female (OR = 5.5; 95% CI: 3.4–7.5), Malay (OR = 3.2; 95% CI: 1.3–8.0), Indian (OR = 4.3; 95% CI: 1.6–12.0) and wearing long sleeves (OR = 2.4; 95% CI: 1.1–5.4) were associated with vitamin D deficiency (Table 2).

**Table 2:** Risk factors associated with Vitamin D deficiency using simple and multiple logistic regression for all participants.

Variables	Vitamin D Deficiency	Vitamin D Sufficiency	Crude OR (95% CI)	p-value	Adjusted OR (95%CI)	p-value
	N = 338 (%)	N = 678 (%)				
**Gender**				<0.001		< 0.001
Female	296 (47)	335 (53)	7.2 (5.1, 10.3)		5.5 (3.4,7.5)	
Male	42 (11)	343 (89)	1.0 (ref)		1.0 (ref)	
**Ethnicity**				<0.001		0.02
Malay	301(36)	545 (64)	3.9 (2.1, 7.5)		3.2 (1.3,8.0)	
Indian	26 (32)	55 (68)	3.4 (1.5, 7.4)		4.3 (1.6,12.0)	
Chinese	8 (8)	65 (92)	1.0 (ref)		1.0 (ref)	
**Body Fat Percentage (%)**						
Obese (≥25^m^; ≥33.8^f^)	103 (41)	147 (59)	1.6 (1.2,2.1)	0.002	1.4 (0.8,2.6)	0.05
Non-Obese (<25^m^;<33.8^f^)	235 (31)	531 (69)	1.0 (ref)		1.0 (ref)	
**School Location**				0.08		0.17
Urban	217 (35)	396(65)	1.3 (0.9, 1.7)		1.3 (0.9, 1.8)	
Rural	121 (30)	282 (70)	1.0 (ref)		1.0 (ref)	
**Cloud Cover (mean in okta)**	6.85 ± 0.35	6.87 ± 0.33	0.8 (0.6, 1.2)	0.33	-	-
**Skin Pigmentation**				0.03		0.36
Type 2	92(34)	177(66)	0.6 (0.3,1.2)		0.6 (0.2, 1.4)	
Type 3	140(36)	249 (64)	0.6 (0.3,1.3)		0.7 (0.3, 1.8)	
Type 4	79(30)	189(70)	0.5 (0.2,1.0)		0.6 (0.2, 1.5)	
Type 5 & 6	11 (20)	45 (80)	0.3 (0.1,0.7)		0.4 (0.1, 1.2)	
Type 1	16 (47)	18 (53)	1.0 (ref)		1.0 (ref)	
**Skin Area Most Commonly Exposed**				<0.001		0.57
**Hands and feet only**	202(50)	199 (50)	3.4(1.9, 4.5)		0.6 (0.1, 3.5)	
Wears short sleeves	109(26)	315(74)	1.2 (0.3, 4.3)		0.5 (0.1, 2.8)	
Wears shorts and T-shirt with short sleeves	24(13)	154(87)	0.5 (0.1, 2.0)		0.4 (0.1,2.4)	
Only wears shorts	3 (23)	10 (77)	1.0 (ref)		1.0 (ref)	
**Timing of Outdoor Activities**				0.008		0.30
7am-10am	62 (41)	90 (59)	2.1 (1.1, 4.1)		1.5 (0.7, 3,3)	
2pm-4pm	76(42)	107 (58)	2.2 (1.1, 4.2)		1.5 (0.7, 3.2)	
4pm-7pm	143(31)	319 (69)	1.4 (0.7, 2.5)		1.1 (0.5, 2.2)	
10am-2pm	15 (25)	46 (75)	1.0 (ref)		1.0 (ref)	
**Duration of Outdoor Activities During Weekdays**				0.06		0.25
< 30 min	112 (41)	160 (59)	1.4 (1.0, 2.0)		1.0 (0.7, 1.7)	
30min-1 hour	59 (31)	129(69)	0.9 (0.6, 1.4)		0.8 (0.5, 1.4)	
1 hour-2 hours	65(26)	185(74)	0.7 (0.5, 1.0)		0.7 (0.4, 1.1)	
>2 hours	102(33)	204(67)	1.0 (ref)		1.0 (ref)	
**Duration of Outdoor Activities During Weekend**				<0.001		0.12
< 30 min	70(48)	75 (52)	2.6 (1.8,3.9)		1.7(1.0,2.9)	
30min-1 hour	81(40)	121(60)	1.9 (1.3, 2.7)		1.3(0.8, 2.2)	
1 hour-2 hours	79(30)	182(70)	1.2 (0.9, 1.7)		0.9 (0.6, 1.4)	
>2 hours	108(27)	300(73)	1.0 (ref)		1.0 (ref)	
**How Often Do You Wear Sunscreen?**				0.27		
Sometimes	113 (36)	199(64)	1.2 (0.9,1.6)		-	-
Always	19 (38)	31(62)	1.3 (0.7,2.4)		-	-
Never	206 (32)	448 (68)	1.0(ref)		-	-
**How Often Do You Wear a Shirt with Long Sleeves that Cover Your Shoulders?**				<0.001		0.05
Sometimes	149(25)	440(75)	1.3 (0.8,2.3)		1.5 (0.7, 3.2)	
Always	169(52)	156 (48)	4.4 (2.6,7.5)		2.4 (1.1,5.4)	
Never	20 (20)	82(80)	1.0 (ref)		1.0 (ref)	
**How Often Do You Wear a Hat/Veil?**				0.05		0.39
Sometimes	103(28)	271(72)	0.6 (0.5,0.8)		1.1 (0.7, 1.6)	
Always	5(14)	32(86)	0.3 (0.1, 0.7)		0.4 (0.1, 1.6)	
Never	230 (38)	375 (62)	1.0 (ref)		1.0 (ref)	
**How Often Do You Stay in the Shades or Under an Umbrella?**				0.50		
Sometimes	230 (33)	460 (67)	0.9 (0.6,1.3)		-	-
Always	55(30)	126(70)	0.8 (0.5, 1.2)		-	-
Never	53(37)	92 (63)	1.0(ref)		-	-
**How Often Do You Wear Sunglasses?**				0.02		0.38
Sometimes	99(28)	249 (72)	0.7 (0.5, 0.9)		1.9 (0.6, 1.3)	
Always	8(24)	25(76)	0.6 (0.2, 1.3)		1.5 (0.2,1.4)	
Never	231(36)	404 (63)	1.0 (ref)		1.0 (ref)	

A univariable analysis of female participants showed that, being Malay (OR = 6.7; 95% CI: 3.1–14.4) or, Indian (OR = 4.5; 95% CI: 1.8–11.3), having exposed hands and feet (OR = 3.9; 95% CI: 2.3–6.7), engaging in outdoor activities from 7am to 10am (OR = 2.6; 95% CI: 1.2–5.6), participating in less than 30 minutes of outdoor activities during the weekdays (OR = 1.3; 95% CI: 0.9–2.0) and during the weekend (OR = 2.2; 95% CI: 1.4–3.6) and always wearing long sleeves (OR = 3.7; 95% CI: 1.9–7.2) were associated with vitamin D deficiency. After controlling for other factors, Malay female participants were found to have six times the risk of vitamin D deficiency than their Chinese counterparts (OR = 6.2; 95% CI: 2.0–18.5) while Indian female participants were found to have five times the risk compared to Chinese participants (OR = 5.9; 95%CI: 1.8–19.3) (Table 3).

**Table 3:** Associated factors of vitamin D deficiency by single and multiple logistic regression among female participants.

Variables	Vitamin D Deficiency	Vitamin D Sufficiency	Crude OR (95% CI)	p-value	Adjusted OR (95%CI)	p-value
	N (%)	N (%)				
**Ethnicity**				<0.001		0.005
Malay	267 (51)	254 (49)	6.7 (3.1, 14.4)		6.2 (2.0, 18.5)	
Indian	21 (41)	30 (59)	4.5 (1.8, 11.3)		5.9 (1.8, 19.3)	
Chinese	8 (14)	51 (86)	1.0 (ref)		1.0 (ref)	
**Body Fat Percentage (%)**						
Obese (≥25^m^; ≥33.8^f^)	89 (46)	89 (54)	1.2 (0.8, 1.7)	0.33	-	-
Non-Obese (<25^m^;<33.8^f^)	207 (50)	246 (50)	1.0 (ref)			
**School Location**				0.81	-	-
Urban	190 (47)	212 (53)	1.0 (0.8, 1.4)			
Rural	106 (46)	123 (54)	1.0 (ref)			
**Cloud Cover (mean in okta)**	6.86± 0.31	6.88 ± 0.31	-	0.36	-	-
**Skin Area Most Commonly Exposed**				<0.001		0.42
Hands and Feet Only	197 (55)	160 (45)	3.9 (2.3, 6.7)		1.0 (0.4, 2.5)	
Wears Short Sleeves	79(41)	112 (59)	2.2 (1.2, 4.0)		0.7 (0.3, 1.8)	
Wear shorts and T-shirt with short sleeves& only wear shirt	20(24)	63(76)	1.0 (ref)		1.0 (ref)	
**Timing of Outdoor Activities**				0.03		0.32
7am-10am	54(57)	40 (43)	2.6 (1.2, 5.6)		1.3 (0.5, 3.1)	
2pm-4pm	70 (53)	62 (47)	2.2 (1.0, 4.5)		1.3 (0.5, 2.9)	
4pm-7pm	125(44)	158 (56)	1.5 (0.8, 3.0)		0.9 (0.4, 2.0)	
10am-2pm	14 (34)	27 (66)	1.0 (ref)		1.0 (ref)	
**Duration of Outdoor Activities During Weekdays**				0.08		0.25
< 30 min	102(54)	87 (46)	1.3(0.9, 2.0)		1.3 (0.8, 2.1)	
30min-1 hour	52 (44)	66(56)	0.9 (0.6, 1.4)		0.9 (0.5, 1.5)	
1 hour-2 hours	58 (40)	86 (80)	0.8 (0.5, 1.2)		0.7 (0.4, 1.3)	
>2 hours	84 (47)	96(53)	1.0 (ref)		1.0 (ref)	
**Duration of Outdoor Activities During Weekend**				0.003		0.37
< 30 min	63(61)	40 (39)	2.2 (1.4, 3.6)		1.6 (0.9,3.0)	
30min-1 hour	71 (51)	67(49)	1.5 (1.0, 2.3)		1.2 (0.7,2.0)	
1 hour-2 hours	73(41)	103(59)	1.0 (0.7, 1.5)		1.0 (0.6, 1.6)	
>2 hours	89(42)	125(58)	1.0 (ref)		1.0 (ref)	
**How Often Do You Wear Sunscreen?**				0.55	-	-
Sometimes	18(39)	28(61)	0.7 (0.4, 1.3)			
Always	111(47)	124 (53)	1.0 (0.7, 1.4)			
Never	167 (47)	183(53)	1.0 (ref)			
**How Often Do You Wears a Shirt with Long Sleeves that Cover Your Shoulders?**				<0.001		0.14
Sometimes	165(56)	131 (44)	3.7 (1.9, 7.2)		2.3 (0.9,5.8)	
Always	118(42)	166 (58)	2.1 (1.1, 4.1)		1.56 (0.7, 3.7)	
Never	13 (26)	38 (74)	1.0 (ref)		1.0 (ref)	
**How Often Do You Wear a Hat/Veil?**				0.20		0.44
Sometimes	2(18)	9 (82)	0.2 (0.1, 1.1)		0.4 (0.1, 2.0)	
Always	81(47)	93 (53)	1.0 (0.7, 1.4)		1.1 (0.7, 1.7)	
Never	213 (48)	233 (52)	1.0 (ref)		1.0 (ref)	
**How Often Do You Stay in Shades or Under an Umbrella?**				0.24		0.27
Sometimes	52(43)	70(57)	1.2 (0.8, 1.8)		0.5 (0.3, 0.9)	
Always	196 (47)	225(53)	1.6 (0.9, 2.8)		0.5 (0.3, 0.9)	
Never	48 (55)	40 (45)	1.0 (ref)		1.0 (ref)	
**How Often Do You Wear Sunglasses?**				0.29	-	-
Sometimes	6(35)	11(65)	0.6 (0.2, 1.6)			
Always	83(44)	108(56)	0.8 (0.6, 1.1)			
Never	207 (49)	216 (51)	1.0 (ref)			

## Discussions

In this study, the prevalence of vitamin D deficiency was considerable (33%), despite Malaysia being located near the equator at latitude 3.13°N, 101.7°E and having abundant sunlight throughout the year. However, the prevalence of vitamin D deficiency is higher in other parts of Southeast Asia, such as in Jakarta (75%) [43] and Vietnam (48–53%) [44]. Also the prevalence of vitamin D deficiency among the girls in this study was lower (47%) than found among adolescent girls in Beijing[45] and New Delhi[46], which was reported to be almost 90% in both cases.

From the multivariate analysis, female had 5.5 times higher risk of vitamin D deficiency compared to male although more female (73%) had fairer skin (skin pigmentation Type 1 to Type 3) compared to male (60%), p<0.001. This higher risk could be contributed by 57% of the female wore concealing clothes that only expose the hands and feet compared to 11% male. The usage of sunscreen and umbrella in female were also higher compared to male. A study in northern India also showed boys had significantly (p = 0.004) higher vitamin D concentrations than girls [47]. However, a study in southwest England in the UK [23] involving 4393 adolescents (49% girls) showed no association between gender and low vitamin D. A similar observation was reported for adolescent girls and boys in Boston, United States (p-value = 0.33) [2].

Consistent with other research, this study found ethnicity a strong predictor of vitamin D deficiency [23–24] i.e. Indian participants had four times the risk and Malay had three times the risk of vitamin D deficiency compared to Chinese. It has also been noted that the vitamin D level was lower in Hispanics and African Americans in Boston [2], Maori children in New Zealand [24] and non-Caucasians in Southwest England [23]. These associations were present even after adjustment for other risk factors (gender, environmental, cutaneous, sun-exposure and sun-protective behaviour). Despite finding that ethnicities with darker skin colour were more likely to have vitamin D deficiency, this study found that skin pigmentation in itself was not a significant risk after adjusting for other risk factors. In fact, we noted that those with Type 4 and Type 5 skin pigmentation had higher (although not significant) vitamin D level compared to those with fairer skin pigmentation. We further evaluate the factors that may have contributed to this biologically plausible result, including gender, cloud cover, school residence, sun-exposure and sun-protective behaviours (area of skin exposure and duration of sunlight exposure). We found no significant difference in all the above factors except for gender (results not shown). The lower vitamin D level among the fairer skin participants may be explained by the higher number of females with fairer skin pigmentation (73%) and the factors that lead to the findings that females had 5.5 times higher risk of vitamin D deficiency as discussed in detail earlier. Other potential explanation for this result is that factors such as genetic variants in vitamin D receptor gene expression in different ethnicities may influence the vitamin D level, as seen in a number of studies [48–50]. Vitamin D receptor expression and its transactivation of the human cathelicidin antimicrobial protein (CAMP) has been shown to be influenced by FokI genotype and, together with ethnicity, influence 1,25(OH)2D3-elicited CYP24A1 expression and affect vitamin D level [48]. One other potential explanation involves the presence of single nucleotide polymorphisms (SNP) located on the vitamin D receptor (VDR) that may be associated with vitamin D levels [51]. One of the SNPs in the VDR gene which is associated with vitamin D levels is the rs1544410 SNP, located in the intronic region (intron 8 near the 3A end). Malaysian adolescents who has AA genotype of rs 1544410 or known as BsmI SNP had significantly lower vitamin D level, higher risk of vitamin D deficiency and insulin resistance [52].

One other potential explanation for the discrepancy between skin colour/pigmentation and vitamin D level may be explained by the recent discovery of the alternative pathway of vitamin D activation. This alternative pathway, which has been demonstrated either in-vitro or in-vivo (through steroidogenic enzymes cytochrome P450scc (CYP11A1) and CYP27B1 (1α-hydroxylase) in the serum, adrenal gland and placenta apart from the epidermal keratinocytes as the main site for the photo-induced formation of vitamin D suggest that other factors may influence vitamin D level besides skin pigmentation [53–55]. This alternative pathway has broken the dogma of vitamin D3 was solely activated through the sequential hydroxylations by CYP27A1 at C25, and by CYP27B1 at C1 [55]. Another possible reason as to why skin pigmentation did not show a significant effect on the vitamin D level could be the small number of dark skinned adolescents in this study.

Diet may also affect the level of serum 25-hydroxyvitamin D [56]. Perhaps the difference in dietary pattern between the ethnic groups [57] may have contributed to the different level of 25hydroxyvitaminD level. However, we did not include diet history in this analysis and this is one of the limitation of our study. Further study are needed to explore the influences of diet pattern among the different ethnic groups and their level of vitamin D.

Obese children are at higher risk of vitamin D deficiency because of sequestration of vitamin D in the fat tissue, as shown in a number of studies [5, 58–61]. The low vitamin D level in obese children may also be due to their more sedentary lifestyle and lower amount of sun exposure [45]. In this study, obese participants had a significantly lower mean vitamin D level than their non-obese counterparts. However, after combining this factor with other risk factors, body fat percentage was found not to be associated with vitamin D deficiency.

The mean cloud cover in Malaysia was high (defined as more than 5 octa), both in the urban and rural areas as investigated by this study. Cloud cover greater than 5 octa has been shown to reduce the amount of biologically effective ultraviolet radiation for pre-vitamin D_3_ production [18]. However, despite having greater cloud cover, the mean vitamin D level among the participants in the rural area was higher than among those in the urban area, thus cloud cover was found not to be associated with a low vitamin D level. This again shows that the cause of a low vitamin D level is multifactorial.

It has been found that an increase in exposure to sunlight occurs by increasing the duration of outdoor activities and this in turn increases vitamin D synthesis [9]. According to a previous study [2] on the prevalence of vitamin D deficiency in healthy adolescents presented for primary care consultation, a physical activity level higher than 7 hours per week is associated with a 22% higher serum 25-hydroxy vitamin D concentration. Studies in New Delhi [46] and Beijing [45] also show that the duration of sun exposure influenced the level of vitamin D. This present study is consistent to some extent with other studies [2, 13, 62] as the results showed that the mean vitamin D level increased when the duration of outdoor activities both during weekdays and the weekend increased. However, this study also found that the mean vitamin D level plateaued at 2 hours of sun exposure during weekdays and the weekend, where there was no further increase in vitamin D level beyond the 2-hour duration of sun exposure.

Other modifiable risk factors such as the timing of outdoor activities and percentage of skin exposed to sunlight also showed a similar pattern, whereby they were significantly associated with vitamin D deficiency in the univariate analysis, but the associations disappeared (were not significant) in the saturated model. However, the genetic predisposition, e.g. gender and ethnicity may have modified the response to sun exposure and this may explain why the sun exposure factors were not significantly different when combined with other risk factors. This postulation warrants further study, especially in relation to the issue of vitamin D gene expression. Furthermore, the findings in this study may have been influenced by the inaccuracy in the reporting sun-exposure activities (no direct observations) by these participants because they usually do not have regular and predictable patterns of behavior in terms of the timing and duration of outdoors activities. The results may also have been affected by recall bias because the questionnaires relied on self-reported sun-protective and sun-exposure behaviours and there were no direct observations conducted by the researchers.

Sun-protective behaviour, such as using sunscreen, staying in the shade or under an umbrella [16], wearing a hat or veil and wearing sunglasses influences the vitamin D status. A previous study on Malaysian adults [17] found a significant positive correlation with vitamin D level and the sun-exposure score and a negative correlation with the sun protection score. However, the participants were from a single ethnicity i.e. Malays, working in an urban area. This study did not detect a significant association between sun-protective behaviours and vitamin D deficiency except for wearing long sleeves. Participants who wore long sleeves were 2.4 times more likely to have vitamin D deficiency. In this study, 60–70% of the participants did not take any specific sun-protective measures, such as wearing sunglasses, hat or veil or using sunscreen. However, the limited categorization of sun-protective behaviour in this study (never, sometimes and always) may have been inadequate to accurately assess the sun-exposure behaviour of the participants. As such, this may have contributed to the findings in the multivariate analysis, i.e. that sun-protective behaviours was not a significant influencing factor. Other factors such as ethnicity may have played a more important role than the sun-protective factors as shown by a study on adults in the United States[16], where sun-protective behaviour such as wearing long sleeves, stay in the shade or using an umbrella were found to be significant only among Caucasians. The study among the female adolescents in United Arab Emirates showed no difference between the vitamin D level and percentage of surface area unexposed to the sun when outdoors [63].

A further analysis of the female participants in this study showed that ethnicity played a major role in the level of vitamin D. Malay and Indian females were six times more likely to have vitamin D deficiency compared to Chinese females. Thus, further studies are warranted to investigate the differences in the dietary and genetic factors that may have contributed to the higher likelihood of vitamin D deficiency among the Malay and Indian adolescent girls.

Nabila et al [7] reported high prevalence of vitamin D deficiency among 13-year-old Malaysian adolescents. The present study is to further understand the possible risk factors (socio-demographic, clinical, environmental and cutaneous) of vitamin D deficiency among the same cohort of Malaysian adolescents when they are around 15-year-old.

Although 17% of the data were missing, mainly due to the incomplete questionnaires, this would not have significantly affected the results because a large number of participants (n = 1016) were recruited for the study. Most of the respondents were Malays, as the majority of students in public schools are of this ethnicity. The combined proportion of other ethnicities was small (<8%) and this may have affected the analysis and driven the results in a skewed direction.

Also, as mentioned above, self-reports on sun-exposure and sun-protective behaviours were relied upon and recall bias may therefore have influenced the results of this study. Direct observations may reduce recall bias. It should also be noted that diet and vitamin D supplementations, although documented, were not included as possible risk factors in this analysis.

As this study only involved one age-group (around 15 years-old), it would be beneficial for future studies to examine wider age groups, such as 13 to 17 year-old adolescents. Future studies could also consider recruiting different types of schools such as vernacular schools to obtain a broader picture about the vitamin D status in Malaysian adolescents from a diverse ethnic background.

In this study, skin pigmentation was found not to be a significant risk factors for vitamin D deficiency, but ethnicity was identified as an important risk factor. This may be due to the differences in the single nucleotide polymorphism and vitamin D receptor gene. As such, further studies to investigate these genetic variants are warranted.

## Conclusions

In conclusion, the prevalence of vitamin D deficiency among Malaysian adolescents was considerable despite the abundant sunlight. Gender, ethnicity and clothing style were important risk factors. Skin pigmentations, environmental factors, sun-exposure and sun-protective behavior were found not to be the significant risk factors for vitamin D deficiency among Malaysian adolescents.

## Supporting information

S1 File (DOCX)
