# Supplementary material for: Risk factors of vitamin D deficiency among 15-year-old adolescents participating in the Malaysian Health and Adolescents Longitudinal Research Team Study (MyHeARTs)
Source: PLoS One. 2018 Jul 19;13(7):e0200736. doi: 10.1371/journal.pone.0200736 (PMC6053195; doi:10.1371/journal.pone.0200736)
Supplement: S1 File — (DOCX) [file pone.0200736.s001.docx]

**Supporting Information**

S1 File

Questionnaire

(A) Demographic Data

| Name |  | | | | | |
| --- | --- | --- | --- | --- | --- | --- |
| IC number or Passport number |  | | | | | |
| Date of Birth |  | | | | | |
| Gender | Male |  | | Female | |  |
| Race | Malay |  | | Chinese | |  |
|  | Indian |  | | Others | |  |
|  | If others, please specify | | | | | |
| Weight (kg) |  | | Height (cm) | |  | |
| Body Mass Index |  | | Body Fat Percentage | |  | |
| Waist Circumference(cm) |  | | Hip circumference (cm) | |  | |
| Name of the School |  | | | | | |
| School Address |  | | | | | |
| Address |  | | | | | |
| Home Telephone |  | | | | | |
| Mobile |  | | | | | |
| Email Address |  | | | | | |
| Nationality | Malaysian |  | | Non-Malaysian | |  |
|  | If non- Malaysian, please specify your nationality | | | | | |

(2) Do you have any medical illness?

| Yes |  | No |  |
| --- | --- | --- | --- |

If Yes,

(a) What kind of medical illness? ---------------------------------

(b) do you take any form of medication?

(B)Skin Pigmentation


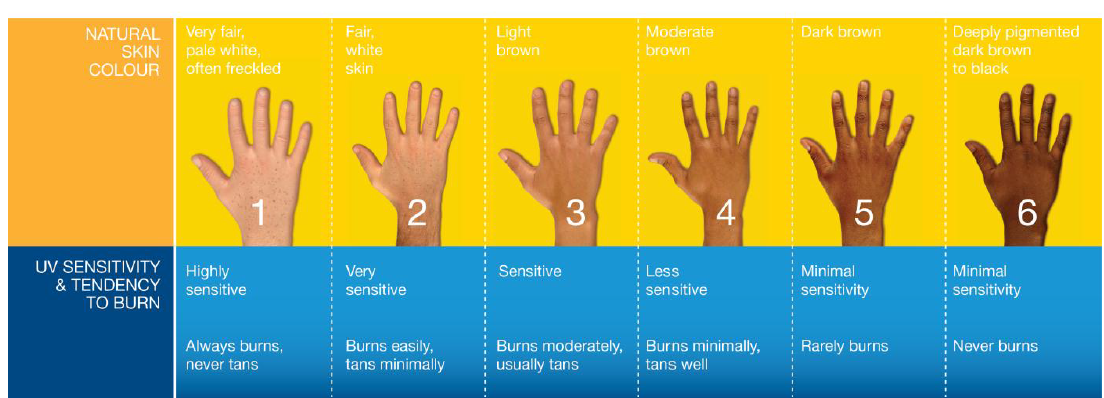


| Please tick mark √ against the skin pigmentation which applicable to you |  |  |  |  |  |  |
| --- | --- | --- | --- | --- | --- | --- |

**(C)Sun Exposure**

For each question listed, please select (tick √) the one answer that is the best response to the question

1. On average, how many hours you spend outdoor per day between 10am and 4pm?

| During Weekdays (mon- fri) | | During weekend (sat- sun) | |
| --- | --- | --- | --- |
| ≤ 30 minutes |  | ≤ 30 minutes |  |
| 31min-1 hours |  | 31min-1 hours |  |
| 1-2 hours |  | 1-2 hours |  |
| > 2 hours |  | > 2 hours |  |

2. When do you go out for outdoor activities?

| 7am-10am |  |
| --- | --- |
| 10am-2pm |  |
| 2pm-4pm |  |
| 4pm-7pm |  |

3. Please tick ✓ against the exposed area to sunlight which applicable to you

|  |  |  |  |
| --- | --- | --- | --- |
|  |  |  |  |

1. For the following questions, think about what you do when you are outside during a sunny day.

|  | Never | Sometimes | Always |
| --- | --- | --- | --- |
| 1. How often do you wear sunscreen |  |  |  |
| 1. How often do you wear a shirt with long sleeves that cover your shoulders? |  |  |  |
| 1. How often do you wear a hat? |  |  |  |
| 1. How often do you stay in the shade or under an umbrella? |  |  |  |
| 1. How often do you wear sunglasses |  |  |  |

(E) Do you take any vitamin D supplements?

| Yes |  | No |  |
| --- | --- | --- | --- |

If Yes, please specify dosage of vitamin D per day …………………..

**References**

1. Glanz K, Yaroch AL, Dancel M, Mona Saraiya, Lori A. Crane, David B. Buller, et al. Measures of Sun Exposure and Sun Protection Practices for Behavioral and epidemiologic research. Arch Dermatology. 2008;144(2),217-222.
2. Fitzpatrick TB. The validity and practicality of sun-reactive skin types I through VI. Arch Dermatology. 1998;124(6),869-898

Borang Soal Selidik

(A) Data Demografi

| Nama |  | | | | | |
| --- | --- | --- | --- | --- | --- | --- |
| Nombor Kad Pengenalan atau nombor passport |  | | | | | |
| Tarikh Lahir |  | | | | | |
| Jantina | Lelaki |  | | Perempuan | |  |
| Bangsa | Melayu |  | | Cina | |  |
|  | Indian |  | | Lain-lain | |  |
|  | Kalau bangsa lain-lain, sila nyatakan | | | | | |
| Berat (kg) |  | | Tinggi (cm) | |  | |
| Index Jisim Badan |  | | Peratusan Lemak Badan | |  | |
| Lilitan Pinggang (cm) |  | | Lilitan Pinggul (cm) | |  | |
| Nama Sekolah |  | | | | | |
| Alamat Sekolah |  | | | | | |
| Alamat Rumah |  | | | | | |
| Number Telefon Rumah |  | | | | | |
| Number Handphone |  | | | | | |
| Alamat e-mel |  | | | | | |
| Kerakyatan | Rakyat Malaysia |  | | Bukan rakyat Malaysia | |  |
|  | Sekiranya bukan rakyat Malaysia, sila nyatakan dari negara mana | | | | | |

(2) Adakah anda menghidapi penyakit apa-apa?

| Ada |  | Tidak |  |
| --- | --- | --- | --- |

Jika ada,

(a) Apa Jenis Penyakit Perubatan? ---------------------------------

(b) Adakah anda mengambil sebarang ubat?

(B) Pigmentasi Kulit


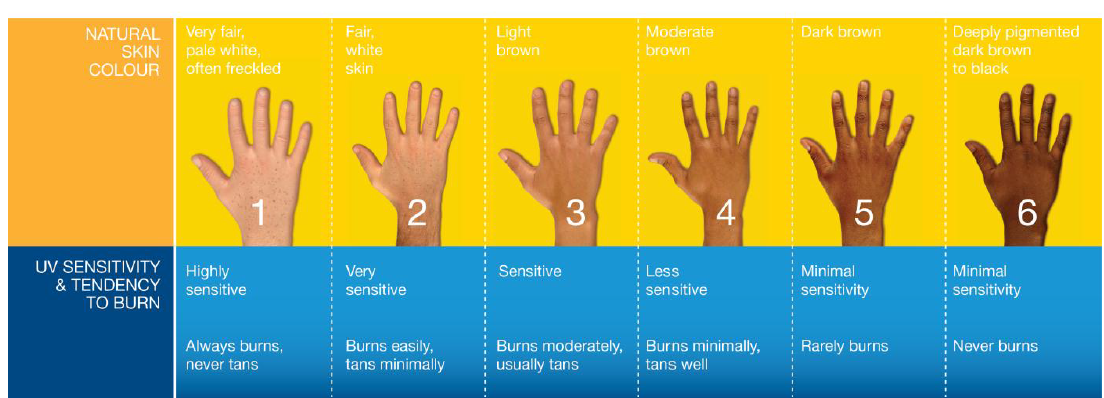


| Sila tandakan √ terhadap pigmentasi kulit anda |  |  |  |  |  |  |
| --- | --- | --- | --- | --- | --- | --- |

**(C)Pendedahan Matahari**

Bagi setiap soalan yang disenaraikan, sila pilih (tandakan ✓) jawapan yang paling sesuai dengan anda

1. Secara purata, berapa jam anda di luar rumah dari pukul 10 pagi hingga empat petang?

| Semasa Hari-Hari Bekerja (Isnin hingga Jummat) | | Semasa Hujung Minggu ( Sabtu hingga Ahad) | |
| --- | --- | --- | --- |
| ≤ 30 minit |  | ≤ 30 minit |  |
| 31minit-1 jam |  | 31minit-1 jam |  |
| 1-2 jam |  | 1-2 jam |  |
| > 2 jam |  | > 2 jam |  |

2. Bila anda keluar untuk aktiviti luar?

| 7am-10am |  |
| --- | --- |
| 10am-2pm |  |
| 2pm-4pm |  |
| 4pm-7pm |  |

3. Sila tandakan ✓ terhadap bahagian badan anda yang terdedah kepada cahaya matahari semasa anda keluar daripada rumah?

| 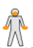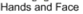 |  |  |  |
| --- | --- | --- | --- |
|  |  |  |  |

1. Untuk soalan-soalan berikut, apa yang anda lakukan semasa anda berada di luar rumah pada hari yang cerah ?

|  | Tidak Pernah | Kadang-kala | selalu |
| --- | --- | --- | --- |
| 1. Berapa kerap anda memakai “sunblock”? |  |  |  |
| 1. Berapa kerap anda memakai baju yang berlengan panjang? |  |  |  |
| 1. Berapa kerap anda memakai topi ? |  |  |  |
| 1. Berapa kerap anda berada di bawah tempat teduh atau di bawah payung? |  |  |  |
| 1. Berapa kerap anda memakai “sun glasses”? |  |  |  |

(E) Adakah anda mengambil vitamin D tambahan?

| Ada |  | Tidak |  |
| --- | --- | --- | --- |

Jika ya, sila nyatakan dos vitamin D yang diambil setiap hari …………………..

**References**

1. Glanz K, Yaroch AL, Dancel M, Mona Saraiya, Lori A. Crane, David B. Buller, et al. Measures of Sun Exposure and Sun Protection Practices for Behavioral and epidemiologic research. Arch Dermatology. 2008;144(2),217-222.
2. Fitzpatrick TB. The validity and practicality of sun-reactive skin types I through VI. Arch Dermatology. 1998;124(6),869-898
